# Risk factors and prevention of posttraumatic stress disorder in Intensive Care Unit patients

**DOI:** 10.1192/j.eurpsy.2022.1736

**Published:** 2022-09-01

**Authors:** I. Caldas, S. Vieira, I. Vidó

**Affiliations:** Centro Hospitalar Psiquiátrico de Lisboa, Psiquiatria Geral, Lisboa, Portugal

**Keywords:** prevention, PTSD, risk factor, ICU

## Abstract

**Introduction:**

Post-traumatic stress disorder (PTSD) is associated with exposure to an actual death or serious injury threatening event , as is the example of an Intensive Care Unit (ICU) patient, and it is characterized by dissociative, avoidance, cognitive and mood symptoms. (1) It is known that ICU patients may develop PTSD with an incidence rate of 10%. (2)

**Objectives:**

Comprehend the correlation between PTSD development and ICU care and its risk factors and ways of prevention.

**Methods:**

The authors conducted a literature review by searching the Pubmed database using the keywords PTSD; ICU; Risk Factors; Prevention.

**Results:**

The studies show that the risk factors are associated to: Intensive care like mechanic ventilation, sedation (like using midazolam, lorazepam or opioid); individual’s characteristics like being younger than 50 years old, personal history of depression, feminine gender and lower levels of cortisol, and experiencing cognitive alterations, as hallucinations, delirium, amnesia and delirant memory, or anxiety while under ICU care. (1,3,4,5) As a form of prevention non pharmacological measures are the most consensual. Pharmacologic hypothesis should be applied in the first 6 hours of trauma and could be hydrocortisone, as it is thought to be a protective factor for memory consolidation, but the conclusions are not consistent.(6)

**Conclusions:**

There are a lot of people that develop PTSD in the ICU context who are not diagnosed and therefore not treated. In this way, it is necessary to identify the patients with more risk factors, apply the non-pharmacological measures and evaluate the person after discharge.

**Disclosure:**

No significant relationships.

